# Supplementary material for: Breastfeeding complexities and sociocultural barriers in the context of preventing perinatal transmission of HIV: A descriptive phenomenology in Northern Ghana
Source: PLoS One. 2025 Jul 1;20(7):e0327353. doi: 10.1371/journal.pone.0327353 (PMC12212534; doi:10.1371/journal.pone.0327353)
Supplement: Attachment — Submitted filename: PONE-D-24-40482.pdf [file pone.0327353.s003.pdf]

PLOS ONE

Indigenous Breastfeeding Beliefs and Practices in Rural Northern Ghana: A Study Among HIV-positive Mothers

--Manuscript Draft--

**Manuscript Number:**	PONE-D-24-40482
**Article Type:**	Research Article
**Full Title:**	Indigenous Breastfeeding Beliefs and Practices in Rural Northern Ghana: A Study Among HIV-positive Mothers
**Short Title:**	Indigenous Breastfeeding Beliefs and Practices in Rural Northern Ghana
**Corresponding Author:**	Awinaba Amoah Adongo, PhD Candidate<br>Kwame Nkrumah University of Science and Technology<br>kumasi, kumasi GHANA
**Keywords:**	Customary and traditional beliefs; Exclusive breastfeeding; HIV exposed infants; HIV-positive pregnant and postpartum mothers
**Abstract:**	Purpose: Although World Health Organization policies aim to increase exclusive breastfeeding and replacement breastfeeding for HIV-exposed infants, few studies consider the social and cultural barriers faced by HIV-positive mothers in rural health facilities in Ghana. This study examines barriers to exclusive and replacement breastfeeding among HIV-positive mothers in rural areas, focusing on how cultural beliefs affect adherence to breastfeeding guidelines.<br>Methods: The study employed qualitative research methods in two rural community health facilities, involving 32 respondents. Data were collected through in-depth interviews and informal conversations, transcribed verbatim, and analyzed using Colaizze's descriptive phenomenology data analysis with the NVivo 12 software.<br>Results: The study identified specific sociocultural barriers to preventing mother-to-child transmission of HIV in rural communities. Key barriers include “infants water feeding” due to the belief that breastmilk makes them thirsty, using "zoomkoom" as an alternative supplement, and beliefs about the toxicity of foremilk, referred to as “yia biisum la sagum mee.” For first-time mothers, foremilk is tested by placing insects in it; if the insects survive, the milk is considered safe, but if they die, the milk is deemed hazardous and treated locally before nursing. Factors influencing exclusive breastfeeding and replacement breastfeeding among HIV-positive mothers were multifaceted. These contributing factors ultimately shape exclusive breastfeeding and replacement breastfeeding among HIV-positive mothers.<br>Conclusions: The study enhances the understanding of sociocultural barriers to preventing mother-to-child transmission of HIV and recommends designing culturally adaptive policies that include acceptable community-based breastfeeding intervention programmes in rural settings.<br>Keywords: Customary and traditional beliefs; Exclusive breastfeeding; HIV exposed infants;HIV-positive pregnant and postpartum mothers.
**Order of Authors:**	Awinaba Amoah Adongo, PhD Candidate
	Kofi Osei Akuoko, PhD
	Jonathan Mensah Dapaah, PhD
	Esmeranda Manful, PhD
**Additional Information:**	
**Question**	**Response**
**Financial Disclosure**<br><br>Enter a financial disclosure statement that describes the sources of funding for the work included in this submission. Review the submission guidelines for detailed	The author(s) received no specific funding for this work.

Powered by Editorial Manager® and ProduXion Manager® from Aries Systems Corporation

requirements. View published research articles from *PLOS ONE* for specific examples.<br><br>This statement is required for submission and **will appear in the published article** if the submission is accepted. Please make sure it is accurate.<br><br>**Funded studies**<br>Enter a statement with the following details:<br>• Initials of the authors who received each award<br>• Grant numbers awarded to each author<br>• The full name of each funder<br>• URL of each funder website<br>• Did the sponsors or funders play any role in the study design, data collection and analysis, decision to publish, or preparation of the manuscript?<br><br>Did you receive funding for this work?	
**Competing Interests**<br><br>Use the instructions below to enter a competing interest statement for this submission. On behalf of all authors, disclose any competing interests that could be perceived to bias this work—acknowledging all financial support and any other relevant financial or non-financial competing interests.<br><br>This statement is required for submission and **will appear in the published article** if the submission is accepted. Please make sure it is accurate and that any funding sources listed in your Funding Information later in the submission form are also declared in your Financial Disclosure statement.<br><br>View published research articles from *PLOS ONE* for specific examples.	No competing interest

**NO authors have competing interests**<br><br>Enter: *The authors have declared that no competing interests exist.*<br><br>**Authors with competing interests**<br><br>Enter competing interest details beginning with this statement:<br><br>*I have read the journal's policy and the authors of this manuscript have the following competing interests: [insert competing interests here]*<br><br>* typeset	
**Ethics Statement**<br><br>Enter an ethics statement for this submission. This statement is required if the study involved:<br><br>• Human participants<br>• Human specimens or tissue<br>• Vertebrate animals or cephalopods<br>• Vertebrate embryos or tissues<br>• Field research<br><br>Write "N/A" if the submission does not require an ethics statement.<br><br>General guidance is provided below. Consult the submission guidelines for detailed instructions. **Make sure that all information entered here is included in the Methods section of the manuscript.**	This PhD thesis was ethically approved by the Committee on Human Research, Publication, and Ethics, School of Medicine and Dentistry (CHRPE/AP/497/21), and the Regional Directorate of Health Services (RDHS/11/001). Permission to collect data from hospitals was also obtained, and all participants provided written informed consent

**Format for specific study types**<br><br>**Human Subject Research (involving human participants and/or tissue)**<br>• Give the name of the institutional review board or ethics committee that approved the study<br>• Include the approval number and/or a statement indicating approval of this research<br>• Indicate the form of consent obtained (written/oral) or the reason that consent was not obtained (e.g. the data were analyzed anonymously)<br><br>**Animal Research (involving vertebrate animals, embryos or tissues)**<br>• Provide the name of the Institutional Animal Care and Use Committee (IACUC) or other relevant ethics board that reviewed the study protocol, and indicate whether they approved this research or granted a formal waiver of ethical approval<br>• Include an approval number if one was obtained<br>• If the study involved *non-human primates*, add *additional details* about animal welfare and steps taken to ameliorate suffering<br>• If anesthesia, euthanasia, or any kind of animal sacrifice is part of the study, include briefly which substances and/or methods were applied<br><br>**Field Research**<br><br>Include the following details if this study involves the collection of plant, animal, or other materials from a natural setting:<br>• Field permit number<br>• Name of the institution or relevant body that granted permission	
**Data Availability**<br><br>Authors are required to make all data underlying the findings described fully available, without restriction, and from the time of publication. PLOS allows rare exceptions to address legal and ethical concerns. See the PLOS Data Policy and FAQ for detailed information.	No - some restrictions will apply

Powered by Editorial Manager® and ProduXion Manager® from Aries Systems Corporation

A Data Availability Statement describing where the data can be found is required at submission. Your answers to this question constitute the Data Availability Statement and **will be published in the article**, if accepted.<br><br>**Important:** Stating 'data available on request from the author' is not sufficient. If your data are only available upon request, select 'No' for the first question and explain your exceptional situation in the text box.<br><br>Do the authors confirm that all data underlying the findings described in their manuscript are fully available without restriction?	
**Describe where the data may be found in full sentences. If you are copying our sample text, replace any instances of XXX with the appropriate details.**<br><br>• If the data are **held or will be held in a public repository**, include URLs, accession numbers or DOIs. If this information will only be available after acceptance, indicate this by ticking the box below. For example: *All XXX files are available from the XXX database (accession number(s) XXX, XXX.*).<br>• If the data are all contained **within the manuscript and/or Supporting Information files**, enter the following: *All relevant data are within the manuscript and its Supporting Information files.*<br>• If neither of these applies but you are able to provide **details of access elsewhere**, with or without limitations, please do so. For example:<br><br>*Data cannot be shared publicly because of [XXX]. Data are available from the XXX Institutional Data Access / Ethics Committee (contact via XXX) for researchers who meet the criteria for access to confidential data.*<br><br>*The data underlying the results presented in the study are available from (include the name of the third party*	Data cannot be shared publicly due to the confidentiality of participants involved

and contact information or URL).<br>• This text is appropriate if the data are owned by a third party and authors do not have permission to share the data.<br><br>* typeset	
Additional data availability information:	

Powered by Editorial Manager® and ProduXion Manager® from Aries Systems Corporation

Manuscript Click here to access/download;Manuscript;A main Blind manuscript.docx

Full Title: Indigenous breastfeeding beliefs and practices in rural northern Ghana: A study among mothers living with HIV

Title: Breastfeeding complexities and sociocultural barriers in the context of preventing perinatal transmission of HIV: A descriptive phenomenology in Northern Ghana

Corresponding author: Awinaba Amoah Adongo (PhD Candidate)
Department of Sociology and Social Work
Kwame Nkrumah University of Science & Technology, Kumasi-Ghana
email: awinabaadongo@gmail.com
Contact number: +233249122566
ORCID ID: 0000-0002-0261-3295

Professor Kofi Osei Akuoko
Department of Sociology and Social Work
Kwame Nkrumah University of Science & Technology, Kumasi-Ghana
Email: kofa@gmail.com

Professor Jonathan Mensah Dapaah
Department of Sociology and Social Work
Kwame Nkrumah University of Science & Technology, Kumasi-Ghana
Email: mensah@gmail.com

Professor Esmeranda Manful
Department of Sociology and Social Work
Kwame Nkrumah University of Science & Technology, Kumasi-Ghana
Email: manful@gmail.com

Abstract

Purpose: Although World Health Organization policies aim to increase exclusive breastfeeding and replacement breastfeeding for HIV-exposed infants, few studies consider the social and cultural barriers faced by HIV-positive mothers in rural health facilities in Ghana. This study examines barriers to exclusive and replacement breastfeeding among HIV-positive mothers in rural areas, focusing on how cultural beliefs affect adherence to breastfeeding guidelines.

Methods: The study employed qualitative research methods in two rural community health facilities, involving 32 respondents. Data were collected through in-depth interviews and informal conversations, transcribed verbatim, and analyzed using Colaizze's descriptive phenomenology data analysis with the NVivo 12 software.

Results: The study identified specific sociocultural barriers to preventing mother-to-child transmission of HIV in rural communities. Key barriers include "infants water feeding" due to the belief that breastmilk makes them thirsty, using "zoomkoom" as an alternative supplement, and beliefs about the toxicity of foremilk, referred to as *"yia biisum la sagum mee."* For first-time mothers, foremilk is tested by placing insects in it; if the insects survive, the milk is considered safe, but if they die, the milk is deemed hazardous and treated locally before nursing. Factors influencing exclusive breastfeeding and replacement breastfeeding among HIV-positive mothers were multifaceted. These contributing factors ultimately shape exclusive breastfeeding and replacement breastfeeding among HIV-positive mothers.

Conclusions: The study enhances the understanding of sociocultural barriers to preventing mother-to-child transmission of HIV and recommends designing culturally adaptive policies that include acceptable community-based breastfeeding intervention programmes in rural settings.

Keywords: *Customary and traditional beliefs*; *Exclusive breastfeeding*; *HIV exposed infants; HIV-positive pregnant and postpartum mothers.*

Introduction

Preventing mother-to-child transmission (PMTCT) of HIV remains a critical global health challenge, particularly in sub-Saharan African countries where transmission occurs during pregnancy, childbirth, and breastfeeding (Naidoo et al., 2023; Kassie et al., 2023). Despite efforts, the United Nations Children's Fund (UNICEF, 2020) reported approximately 150,000 new child infections under 0-4 years old, falling short of the target of fewer than 20,000 new infections annually. This underscores the ongoing need for significant efforts to achieve the goals of PMTCT programmes.

PMTCT programmes have evolved significantly to reduce mother-to-child HIV transmission, prompting new research approaches to assess progress and challenges in breastfeeding practices among HIV-positive mothers (Kameni et al., 2022; Fassinou et al., 2023). Evaluating PMTCT impact remains challenging due to cultural factors, healthcare settings, and availability of antiretroviral drugs (WHO, 2022). Programme effectiveness hinges on metrics such as MTCT rates and the proportion of exposed children who remain HIV-free at birth and during breastfeeding, necessitating comprehensive understanding from pregnancy through breastfeeding stages (UNAIDS, 2020).

According to WHO guidelines (2016), mothers living with HIV are recommended to receive antiretroviral therapy (ARV) and exclusively breastfeed (EBF) for six months postpartum, followed by complementary feeding unless safe conditions for replacement feeding are assured. These measures aim to reduce ~~mother-to-child transmission (~~MTCT~~)~~ of HIV, with EBF significantly lowering transmission rates during the first six months of an infant's life. However,

adherence to ARV and EBF practices remains low in sub-Saharan African (SSA) countries, posing challenges to effective PMTCT (Mohammed et al., 2023; Koffi et al., 2023). Cultural perceptions of breastfeeding among pregnant and postpartum women further complicate the adoption of exclusive breastfeeding (EBF) and exclusive replacement feeding (ERF) in SSA (Marinda et al., 2017; Koffi et al., 2023).

Several studies have explored cultural practices related to pregnancy and childbirth, food beliefs, traditional harmful practices, and perinatal care among non-HIV-positive women (Andina Diaze and Siles-Gonalez, 2020; Abebe et al., 2021; Abdul et al., 2021; Tesfaye et al., 2022). However, these studies were conducted outside of the specific study area, highlighting a gap in the literature regarding cultural practices related to breastfeeding among HIV-positive pregnant and postpartum women in Northern Ghana.

This descriptive phenomenological study investigates breastfeeding ~~practices among HIV-positive pregnant and postpartum women in rural communities in Northern Ghana~~, specifically examining adherence to exclusive breastfeeding (EBF) and exclusive replacement feeding (ERF) during the first six months postpartum ~~(Alhazmi & Kaufmann, 2022). The study aims to inform policy interventions aimed at eliminating mother-to-child transmission (MTCT) of HIV during the postpartum period.~~

Theoretical Framework

Phenomenological Framework

A phenomenological framework is a structured approach to research and analysis and describes human experiences from the first person. This paradigm is based on phenomenological philosophy

and seeks to comprehend how people perceive, interpret, and make sense of their experiences (Husserl, 1938; Heidegger 1976). Descriptive phenomenology, rooted in the philosophical ideas of Edmund Husserl, emphasizes the meticulous description of lived experiences to uncover the essence of phenomena was used as the theoretical framework for this study. Husserl (1938) argued that phenomenology seeks to explore how individuals perceive and make sense of their experiences, aiming for a pure description devoid of preconceived notions. This approach involves epoché or bracketing, where researchers set aside their biases to understand the phenomena from the participants' perspectives. By doing so, they can capture the invariant structures of the experience.

Colaizzi (1978) expanded on Husserl's ideas by developing a systematic method for data analysis in descriptive phenomenology. Colaizzi's method involves a rigorous process of extracting significant statements, formulating meanings, and organizing these meanings into themes that capture the essence of the experience. This method ensures that the findings remain grounded in the participants' descriptions while providing a structured approach to phenomenological research.

Max van Manen (1996) contributed to the field by emphasizing the importance of reflexivity and the researcher's engagement with the lived experiences of participants. Van Manen's approach integrates both descriptive and interpretive elements, focusing on how individuals interpret their experiences and the meaning they ascribe to them. This approach underscores the role of the researcher in co-creating understanding through deep engagement with the participants' narratives.

Amedeo Giorgi (2009) further refined descriptive phenomenological methods by proposing a systematic approach that remains faithful to Husserlian principles. Giorgi emphasized the importance of maintaining scientific rigor while capturing the richness of human experience. His

method involves a careful analysis of the data to identify essential structures and a commitment to phenomenological reduction, ensuring that the findings are grounded in the participants' lived experiences.

In the context of breastfeeding complexities and sociocultural barriers present significant challenges in the prevention of mother-to-child transmission (PMTCT) of HIV. Descriptive phenomenology was used to study the lived experiences of HIV positive mothers with breastfeeding complications and societal hurdles in preventing HIV transmission from mother to child.

Using Husserl's (1938) principles, researchers begin by bracketing their preconceptions about breastfeeding and HIV transmission to approach the study with an open mind. This process ensures that the participants' descriptions of their experiences are not influenced by the researchers' biases, allowing for a pure exploration of the phenomenon. Colaizzi's (1978) method guided the data analysis, where significant statements from interviews were extracted and organized into themes. For instance, mothers might describe the fear of HIV transmission through breastfeeding, the stigma associated with HIV, and the pressure to adhere to traditional breastfeeding practices. These themes were synthesized to capture the essence of the lived experiences related to breastfeeding and PMTCT.

Van Manen's (1996) emphasis on reflexivity was crucial in this study, as researchers engage deeply with the participants' narratives. Understanding the cultural context and the meanings that mothers and community members attach to breastfeeding practices provided valuable insights into the sociocultural barriers they face. This engagement helps to illuminate how these barriers affect their decisions and experiences regarding breastfeeding and HIV prevention.

Finally, Giorgi's (2009) systematic approach ensures that the study maintains scientific rigor while delving into the complexities of the participants' experiences. By focusing on the essential structures of these experiences, researchers provided a nuanced understanding of the interplay between breastfeeding practices and sociocultural barriers in the context of PMTCT in Northern Ghana.

In conclusion, descriptive phenomenology allows for a comprehensive exploration of the lived experiences of the participants and the meaning they attributed to those experiences. By adhering to the principles and methods outlined by Husserl, Colaizzi, Van Manen, and Giorgi, researchers can uncover the essence of these experiences and provide insights that could inform more effective interventions for the prevention of mother-to-child transmission of HIV.

Research Methods

Study setting and design

The study was conducted in Bongo and Talensi District hospitals in the Upper East Region of Northern Ghana, located 14.8 km and 31 km from the regional capital, respectively. These districts face HIV/AIDS determinants such as high-risk sexual behaviour, traditional marketing, low literacy rates, polygamy, widow inheritance, a nonchalant lifestyle, and migration. The region recorded 5534 teenage pregnancies in 2021. The study area was chosen for their rich cultural values and deprived rural status for the understanding of the phenomenon.

In this qualitative study, a phenomenological design was used to understand the phenomenon in its natural setting. Descriptive phenomenology is a qualitative research methodology aimed at capturing and describing the lived experiences of individuals regarding a specific phenomenon.

Rooted in the philosophical traditions of Edmund Husserl, this approach focuses on understanding the essence of experiences from the first-person perspective, without the influence of the researcher's preconceptions or biases (Moustakas, 1994). In this paper, descriptive phenomenology would involve collecting detailed narratives from mothers about their breastfeeding experiences, especially as they navigate the challenges posed by HIV prevention protocols.

A critical aspect of descriptive phenomenology is the practice of bracketing, epoché, and phenomenological reduction. Bracketing involves the researcher setting aside their own beliefs, judgments, and experiences to view the phenomenon purely from the participants' perspective (Creswell, 2013). This process ensures that the data collected is as close to the lived experience of the participants as possible, free from external interpretations. In this study, the researchers bracketed their own views on breastfeeding and HIV transmission to understand how the participants perceive and experience these issues.

Epoché, closely related to bracketing, is the process by which the researcher suspends all preconceived notions and biases, entering the research with a fresh perspective (Moustakas, 1994). This state of openness allows the researcher to grasp the true essence of the participants' experiences. In this study, the researcher approached each interview without prior assumptions about cultural practices or healthcare barriers, allowing the mothers' authentic voices to emerge.

Phenomenological reduction, another core element of descriptive phenomenology, involves breaking down the experiences into their essential structures. It is a method of analysis that seeks to uncover the fundamental meaning of the experiences described by participants (Colaizzi, 1978; Giorgi, 2009). By focusing on the invariant aspects of these experiences, the researcher can

identify the core themes and essences that define the phenomenon. In this study, reduction would involve analysing the narratives of HIV-positive mothers to distil the essential themes related to breastfeeding complexities and sociocultural barriers, providing a clearer understanding of their lived experiences. Data were collected from April to December 2022 through informal and in-depth interviews with HIV-positive pregnant and postpartum women using PMTCT services to explore sociocultural barriers to exclusive and replacement breastfeeding. The data were analysed using Colaizzi's (1978) descriptive phenomenological technique.

Participants and recruitment criteria

Participants in the study included HIV-positive pregnant and postpartum women who had previously used the PMTCT service at the health facilities in these rural communities. Participants who were HIV-positive but not pregnant or breastfeeding were not allowed to participate in the study. A purposive sampling method was employed to recruit 32 women from the rural community's health facilities. The selection process was guided by the research objective, aiming to include women with diverse experiences related to the phenomenon under investigation. Health facility records and referrals from healthcare providers were used to identify potential participants who met the inclusion criteria. These women were then approached and informed about the study, and those who agreed to participate were included. The purposive sampling strategy ensured that participants with a wide range of experiences were selected to provide rich, in-depth insights into the phenomenon, thereby enhancing the quality and comprehensiveness of the findings (Creswell & Creswell, 2018).

Data collection tool and procedure

Data collection was carried out systematically to ensure both the quality of the data and the comfort and confidentiality of the participants. Information was gathered through informal and in-depth interviews, allowing for a flexible and conversational approach to understanding participants' experiences. The data collection took place from March 28, 2022, to June 28, 2022, followed by participant follow-up and validation from July 22, 2022, to September 23, 2022. These interviews were conducted in an office assigned by the facility's unit head, but the researcher also made accommodations by meeting participants in their preferred locations, such as their homes, school grounds, and convenient hospital locations. This approach helped create a more comfortable and familiar environment for the participants, encouraging open and honest dialogue.

To address the sensitive nature of the study and reduce the risk of stigma, the researcher took great care to ensure privacy during interviews. Participants expressed a strong preference for privacy, and the researcher respected this by selecting discreet interview locations and scheduling sessions at times when participants felt most comfortable and secure. This helped to foster a sense of trust and safety, which was crucial for collecting authentic and detailed information.

Before the interviews began, all participants who agreed to join the study were required to sign or thumbprint the consent form, ensuring they fully understood the purpose of the research and their rights as participants. The consent process included reading the information to those who needed assistance, with ample opportunity for participants to ask questions and clarify any concerns. This thorough consent process was essential in building trust and ensuring ethical standards were maintained throughout the study.

Anonymity was strictly upheld by assigning pseudonyms to all participants, ensuring that their identities remained confidential. Interviews typically lasted between 45 and 60 minutes, providing ample time for participants to express their experiences in detail. The researcher employed a semi-structured interview format, using open-ended questions to guide the conversation while allowing participants the freedom to discuss topics that were most relevant to them. This approach not only ensured the collection of rich, in-depth data but also allowed the researcher to explore emerging themes as they arose.

Interviews were conducted until data saturation was reached, meaning that the researcher continued to gather data until no new information or themes emerged. This approach ensured that the data collection process was thorough and that the findings were representative of the full range of participants' experiences. Also, the researcher took detailed notes and used audio recording (with participants' consent) to capture the nuances of each interview, ensuring the accuracy and reliability of the data collected. This careful and systematic approach to data collection was integral to the study's overall rigor and credibility.

Data analysis

The Colaizzi (1978) method, a popular data analysis technique in descriptive phenomenology, was utilized to analyse the data collected from participants. This method emphasizes a rigorous, structured approach to capturing the true essence of participants' lived experiences. The process began with familiarization, where the researcher read and re-read the interview transcripts multiple times to deeply understand the participants' perspectives and experiences.

Next, the researcher identified significant statements from the transcripts, selecting key phrases or sentences that directly related to the phenomenon being studied. These significant statements were

extracted and organized in a word document template for easy reference. From these statements, meanings were carefully formulated, ensuring that the participants' intended meanings were accurately interpreted without imposing bias or misinterpretation.

The next step involved clustering formulated meanings into themes, where the researcher grouped similar meanings together to create broader, overarching themes that reflected common patterns across participants' experiences. These themes were then used to develop a detailed and exhaustive description of the phenomenon, ensuring that the depth and complexity of participants' experiences were fully represented.

To make the findings more accessible, this comprehensive description was condensed into a concise summary, highlighting the most critical aspects of the phenomenon. Finally, the researcher conducted a validation process by contacting participants via personal visits or phone calls, ensuring that the findings accurately reflected their experiences. This validation step enhanced the credibility and reliability of the study's conclusions. The Colaizzi method provided a transparent and methodical approach to data analysis, ensuring that the research remained grounded in participants' authentic experiences. An example illustrating the development of theme clusters and emergent themes from significant statements has been provided in Table 1.

Table 1 Development of clusters of themes and emergent themes from the significant statements and formulated meanings.

Significant Statements	Formulated Meanings	Theme Clusters	Emergent Themes
"Breastfeeding is something we have always done in our culture."	Breastfeeding is a long-standing practice passed down through generations.	Long-standing practices. Traditional health benefits of breastfeeding.	Breastfeeding as a cultural practice

We believe that giving infants water helps with digestion."	Providing infants with water is a traditional practice believed to aid in digestion	Beliefs it improves infant's health. Ancestral customs and practices.	Traditional infants' water feeding as a cultural practice
"The elder women in the family are very influential in child-rearing practices."	Elder women, particularly mothers-in-law, have a strong influence on child-rearing practices.	Influence of elder women in the family. Respect for elders' advice.	The traditional and customary role of the mother-in-law
"There are times when we struggle to have enough food for everyone."	Food scarcity is a challenge that impacts family well-being.	Challenges in food availability. Psychological impact of food on medication.	Food security

Source: Field Data transcribed (2022)

Trustworthiness/Rigour

To ensure trustworthiness in the qualitative research, the four components outlined by Lincoln and Guba (1985) credibility, transferability, confirmability, and dependability were adequately addressed. Credibility was ensured through audio recording, verbatim transcription, and peer evaluation. Transferability was enhanced by providing detailed descriptions to facilitate application to similar work. Confirmability was maintained using bracketing to avoid personal bias. Dependability was ensured by using audit trails. Accuracy was enhanced through prolonged engagement, involving extended time with respondents in their native culture to understand their behaviour, values, and social relationships in context.

Ethical statement

This PhD thesis was ethically approved by the Committee on Human Research, Publication, and Ethics, School of Medicine and Dentistry (**CHRPE/AP/497/21),** and the Regional Directorate of Health Services **(RDHS/11/001).** Permission to collect data from hospitals were also obtained and all participants provided written informed consent.

Results

Socio-demographic characteristics of the participants

Participants in the study ranged in age from 20, the youngest, to 47, the oldest, with women having between zero and four children. Out of the children tested, 14 were found to be HIV positive, while the HIV status of eight children remained unknown pending test results. Factors contributing to mother-to-child HIV transmission included refusal to attend antenatal care, reject HIV test results, opt for traditional treatments over antiretrovirals, and delay hospital treatment. Only five mothers practiced exclusive breastfeeding, despite widespread knowledge of its benefits. The majority of participants were married, with few being single, widowed, or divorced. Educational backgrounds varied, with one participant having university education, one with polytechnic education, seven with secondary education, seven with junior high education, and four with primary education; others had no formal education. Most participants identified as Christian, with a minority being Muslim, and the majority were engaged in farming, while one was a teacher and another a trader.

Presentation of results

According to the findings of the study, four major themes emerged from the data regarding exclusive breastfeeding practices among HIV-positive pregnant and postpartum women. The emergent themes included breastfeeding as a cultural practice, "traditional infants water feeding" as a cultural practice, the customary of the mother-in-law during breastfeeding, and lack of food. The Table 2 gives a summary of the cluster of themes and emergent themes of the study participants.

Table 2 The emergent and cluster themes

Emergent themes	Cluster of themes

Breastfeeding as a cultural practice	• Breastfeeding as long-standing practices.<br>• Traditional health benefits of breastfeeding.
Traditional infants' water feeding as a cultural practice	• Beliefs it improves infant's health.<br>• Ancestral customs and practices
The traditional and customary role of the mother-in-law	• Influence of elder women in the family.<br>• Respect for elders' advice.
Food security	• Challenges in food availability.<br>• Psychological impact of food on medication

Source: Field Data transcribed (2022)

Theme 1: Breastfeeding as a cultural practice

Breastfeeding as a cultural practice is deeply rooted in tradition, passed down through generations as a long-standing practice. It is highly valued for its health benefits, promoting infant health and strengthening the bond between mother and child.

***Breastfeeding as a long-standing practice*.**

The positive mothers discussed their experiences with breastfeeding as a cultural practice rather than an option for exclusive breastfeeding (EBF) or exclusive replacement feeding (ERF). A woman cannot choose whether or not to breastfeed according to traditional practice. As a mother, you cannot choose any of the alternative feeding options because it is a cultural practice that a woman must breastfeed her baby. One of the health workers said:

> *Breastfeeding in PMTCT, the woman has the option to breastfeed or not breastfeed, but traditionally, the woman has no choice, so you must breastfeed the baby...... If you wean the child in a year, the mother-in-law will object, and other family members will insist that the child be breastfed for at least two years* (Juliet, health worker)

Traditional health benefits of breastfeeding

Traditional health benefits of breastfeeding include providing essential nutrients and antibodies to infants, which boost their immune system and protect against illnesses. Additionally, breastfeeding promotes optimal growth and development, contributing to the overall health and well-being of the baby. A participant said:

> *Breastfeeding helps to keep the baby healthy and strong and you opt not to practice breastfeeding in the traditional family house." Atipoka, HIV-positive mother.*

One of the participants explained why she is unable to practise exclusive breastfeeding due to the social environment in which she lives. Exclusive breastfeeding practise is influenced by farming activities and the nature of child feeding in the community. As a result, only a small number of mothers may practise exclusive breastfeeding.

> *I feed the child whatever food the child can eat. I also give the child water. Many mothers do not do exclusive breastfeeding in this community here because of the farming activities during this season. They will be busy on their farms and will not always be available to breastfeed the child all the times. There is also the belief that you must breastfeed the child as a mother even if you can afford the formular food you cannot choose to do replacement feeding; if you choose to do so, your family members will not understand* (Talenmah, HIV-positive mother).

Theme 2: Traditional infants water feeding" as a cultural practice.

The term "traditional infants water feeding" refers to a cultural practice in northern Ghana where infants are given water forcibly, usually by their mothers or mothers-in-law, particularly in rural communities. This is illustrated in the two subthemes: Beliefs it improves the infant's health and ancestral customs and practices.

Beliefs it improves infant's health

According to the participants this practice is often employed when a child is perceived to be unwell or unwilling to drink. Mothers-in-law believe that when a child cries, it indicates abdominal discomfort, and they prepare boiled herbs and leaves for the child to ingest, aiming to cleanse the intestines and strengthen the immune system.

> *They bathed the child in water boiled with leaves and herbs after I gave birth. They also gave the child water, and the name is "nyuule" [to place the child on a woman's lapse and give water]. This was done to cleanse the child's intestines and strengthen the child. When I became pregnant and gave birth, I was fifteen years old* (Amiamah, HIV-positive mother).

To the mothers, they felt that it is the mothers-in-law make it difficult to practice exclusive breastfeeding. They cannot also prevent them if they want to give water to the child, especially when the is sick. This is what she narrated:

> *Mother-in-law occasionally "nyuules" the child [giving the child water using the traditional method] to make the child strong, and you cannot stop her from doing so. Family members can make it difficult to practise exclusive breastfeeding because they believe we were only giving water and did not die* (Apambila, HIV-positive mother)

Ancestral customs and practices

The practice of giving water to infants is rooted in ancestral customs and my mother-in-law always recommended their daughters-in-law to do so. This is what one of the health workers said:

> *The mothers-in-law do not always want their daughters-in-law to breastfeed exclusively, but when educated, they sometimes understand but are unwilling to allow their daughters-in-law to practise it* (Elizabeth, health worker)

Theme 3: Traditional and customary role of mother-in-law

In traditional societies, mothers-in-law play significant roles during their daughters-in-law's pregnancies and after childbirth. They guide and influence decisions, particularly regarding

feeding practices like exclusive breastfeeding or replacement feeding. Their involvement shapes maternal and child health practices. The two subthemes below illustrate this.

Influence of elder's women in the family

According to the participants, elder women, particularly mothers-in-law, have a strong influence on child-rearing practices. One of the mothers said:

> *My baby was fed with breastmilk and water. I did not breastfeed exclusively because my mother-in-law refused, claiming that giving the child barbal water makes the child healthy and strong* (Abonpoka, HIV-positive mother)

The mothers-in-law do not want their daughters-in-law to practice exclusive breastfeeding because they feel like we are punishing the child. To demonstrate this, one of the health workers said:

> *Exclusive breastfeeding is not commonly practised in this community. Due to the mother-in-perception law's that we are punishing the child by denying him employment, few mothers exclusively breastfeed their infants. The mother-in-law will argue that since you received water from me and you didn't die, why do you want to keep the child away from water?* (Martin, health worker)

The participants also reported that the role of mothers-in-law influences the positive mothers' practise of exclusive breast feeding. The mothers-in-law promote the use of herbs to improve the health of their children. This practise puts MTCT of HIV at risk. This is how Fatima said:

> *The mothers-in-law are the issue because they insist on the child drinking water. When the mother gives birth, the mother-in-law sock herbs and give them to the child to help strengthen the child's body* (Fatima, health worker)

Respect for elders 'advice

One of the health workers said that during ANC, the mothers were educated on the importance of exclusive breastfeeding, and some were aware of its practices; however, they do not want to

disobey their mothers-in-law. The HIV positive mothers listen to mothers-in-law than us. According to one of the participants:

> *"My mother-in-law's advice is highly respected in our household, particularly in this community." (Paulina, Health worker)*

Theme 4: **Food security**

Food security emerged as a critical issue affecting exclusive breastfeeding practices. Participants highlighted the essential role of adequate nutrition in sustaining breastfeeding. The two subthemes below give further illustration on this.

Challenges in food availability

Many reported insufficient food intakes, which they believed directly impacted their milk production. They expressed a belief that better nutrition would increase breast milk production, facilitating exclusive breastfeeding. Inadequate food intake led to concerns about insufficient milk production, prompting supplementation with other foods and liquids alongside breastfeeding. This was stated by one of the participants:

> *There is not enough food for me to eat in order to produce enough breastmilk to feed the baby. I am unable to practise exclusive breastfeeding because breastmilk is insufficient for the baby. So, I will have to feed the baby porridge and "zoomkoom* (Pogbila, HIV-positive mother).

Participants stated that due to a lack of food, "*zoomkoom"* [local drink rich with vitamin C made from water] served as breastmilk for the mother and child. When the baby is crying, the participants indicated that it is fed with "*zoomkoom"*. It was also discovered that when lactating mothers use the "*zoomkoom"*, it produces a large amount of breastmilk for the baby. According to one participant:

If you practise exclusive breastfeeding, you will not be able to give the baby "zoomkoom" again. There is insufficient food for you to eat in order to produce enough breastmilk for the baby. We were told during ANC to practise exclusive breastfeeding, but there is not enough food for you to eat in order to produce enough breastmilk to breastfeed the baby (Asaamah, HIV-positive mother).

Psychological impact of food on medication

One participant stated that food has become a major factor influencing exclusive breastfeeding and medication adherence. There is no food to eat when it is time for her to take the medications, and exclusive breastfeeding would be harmful to the baby. This was what she said:

There is no food to eat before taking the medications, and you want me to exclusively breastfeed my child. Do you want my child to die? Mothers who practise exclusive breastfeeding have a higher socioeconomic status (Asumpoka, HIV-positive mother)

One of the healthcare providers reported that one of the major issues with exclusive breastfeeding in this community is that patients frequently complained about not having enough food to practise exclusive breastfeeding. Lactating mothers revealed that they have begun exclusive breastfeeding but are unable to continue because they do not have enough food to eat in order to produce enough breastmilk for the baby. Some mothers truncated the process along the way and continued the mix feeding. The participant said:

Exclusive breastfeeding is difficult for lactating mothers in this community. They frequently complained about a lack of food, which is the primary source of breastmilk. When asked, some are honest and say they don't practise exclusive breastfeeding due to a lack of food, while others pretend, they do. We don't have any food to give them to enforce it (Ramatu, health worker).

Discussions

The study aimed to explore sociocultural barriers to PMTCT in rural health facilities. It found that exclusive breastfeeding is hindered by factors like *infant water feeding*, using *zoomkoom* as a

breast milk supplement, and cultural perceptions of foremilk. Additionally, cultural practices, the roles of mothers-in-law, and food security significantly impact exclusive breastfeeding among HIV-positive pregnant and postpartum women.

Breastfeeding practices

The PMTCT guidelines recommend that HIV-positive mothers exclusively breastfeed for the first six months before introducing complementary foods or replacement feeding. However, these mothers view breastfeeding as a cultural practice rather than a choice between exclusive breastfeeding (EBF) and exclusive replacement feeding (ERF). Breastfeeding decisions are influenced by family dynamics and cultural practices, with mothers-in-law playing a key role in the decision-making process. The findings indicate that HIV-positive postpartum women consider breastfeeding a cultural practice influenced by several factors: infant water feeding, using *zoomkoom* as a breast milk supplement, and the belief that foremilk is unsafe for newborns. Village elders and families pressure mothers to supplement with *zoomkoom*, believed to enhance breast milk and increase infant weight. Cultural beliefs also hold that breast milk makes infants thirsty and that family members, especially mothers-in-law, should teach breastfeeding. A specific practice, "*yia biisum la sagum mee*," involves testing foremilk with insects to determine its safety for the infant.

HIV-positive mothers reported feeling obligated to breastfeed because formula feeding or exclusive replacement feeding is not considered an option in their community. Most HIV-positive mothers do not use formula feeding, which increases the risk of infant HIV infections due to unsafe environmental and social conditions. These findings align with reports that postnatal HIV transmission during breastfeeding is a major concern in low- and middle-income countries,

particularly in Sub-Saharan Africa, where breastfeeding is the only feasible, safe, and culturally acceptable option (Shetty, 2013; Naidoo et al., 2023). The findings also support the WHO (2017) report that HIV transmission from mother to child typically occurs during pregnancy, childbirth, and breastfeeding. Additionally, the results are consistent with the theory of planned behaviour, which highlights the influence of beliefs, subjective norms, and perceived behavioural control.

However, the findings of this study diverge from the WHO (2020) report, which highlights successful elimination of mother-to-child HIV transmission in countries like Sri Lanka and Thailand through medication adherence and the adoption of formula feeding as an alternative to breastfeeding. This discrepancy may stem from cultural factors that prioritize breastfeeding as the only socially acceptable feeding method and the pervasive poverty in Sub-Saharan Africa, which shape infant feeding practices. Education also plays a crucial role in understanding these differences across regions. The study underscores that adherence to infant feeding guidelines is lacking, and cultural barriers such as using *zoomkoom* to supplement breast milk, cultural beliefs regarding foremilk safety, and perceptions that breast milk induces thirst in infants are undermining efforts to eliminate mother-to-child transmission of HIV, especially in rural communities. The findings support the theory of planned behaviour regarding health beliefs. They recommend promoting education and involving family members in PMTCT programmes to encourage alternative feeding methods and enhance PMTCT outcomes. Additionally, the study suggests expanding PMTCT efforts beyond hospitals to include outreach to families and communities, which could contribute to improvement in this area.

Traditional infants feeding practices

The study highlights the influence of a cultural practice termed "traditional infants water feeding" on exclusive breastfeeding among HIV-positive mothers. Exclusive breastfeeding plays a crucial role in Prevention of Mother-to-Child Transmission (PMTCT) efforts, aligning with WHO and UNAIDS goals to eliminate mother-to-child transmission (MTCT) of HIV and reduce HIV-related morbidity in children. The practice of traditional *infants' water feeding* involves placing the child on the lap to offer water, rooted in the belief that infants are born thirsty and require water from birth. This cultural belief complicates efforts to practice exclusive breastfeeding, as mothers-in-law often advocate for infant water feeding.

Participants in the study disclosed that in instances where a child is sick or reluctant to consume water, mothers or mothers-in-law resort to placing the child on their lap to compel the intake of herbal water. These practices underscore the challenges faced by HIV-positive mothers in adhering to exclusive breastfeeding recommendations due to entrenched cultural beliefs and family influences. This cultural context highlights significant barriers to exclusive breastfeeding, which is essential for PMTCT initiatives. The findings underscore the need for culturally sensitive approaches and community engagement to address these challenges and promote optimal infant feeding practices among HIV-positive mothers. The study reveals that in the study area, many postpartum women find exclusive breastfeeding challenging due to cultural beliefs and practices. For instance, when infants cry persistently, mothers-in-law often attribute this to abdominal pains and advocate for the use of local herbs to cleanse the intestines and boost the immune system. This belief system undermines the practice of exclusive breastfeeding for six months, despite its known benefits and educational efforts promoting its adoption.

These findings echo those of Fauk et al. (2021), who similarly observed that cultural practices influence behaviours that impact HIV transmission, including within spousal relationships. The

World Health Organization (WHO, 2021) also recognizes the pervasive influence of cultural practices in African contexts, noting their implications for HIV prevention from mother to child. This acknowledgment underscores the ongoing challenge of aligning cultural practices with public health recommendations to improve maternal and child health outcomes

Rodriguez et al. (2017) highlighted cultural practices, stigma surrounding HIV, and issues related to disclosure as significant barriers in the ~~Prevention of Mother-to-Child Transmission (~~PMTCT~~)~~ programmes. These factors contribute to challenges in implementing effective HIV prevention strategies among affected populations. Similarly, Malindi, Maputle, and Nemathaga (2022) underscored the impact of traditional, cultural, and religious practices, as well as mixed feeding practices, on increasing the risk of HIV transmission to infants. Their findings emphasize the need for enhanced education aimed at addressing harmful cultural practices to mitigate the risk of Mother-to-Child Transmission (MTCT) of HIV. This is particularly crucial in rural communities where exclusive breastfeeding practices are suboptimal, posing challenges to achieving the UNAIDS 90-90-90 targets for children.

Traditional and customary role of mothers-in-law

Mothers-in-law hold significant influence over reproductive and childcare practices within traditional African societies, stemming from their status as experienced elderly women responsible for family health and decision-making (Falnes et al., 2011). This influence extends to guiding their daughters during pregnancies and childbirth, where they play a pivotal role in instructing on various aspects. Specifically, mothers-in-law often have a decisive say in whether their daughters opt for exclusive breastfeeding or exclusive replacement feeding, highlighting their authority in shaping infant feeding practices within the family dynamic. These findings corroborate the

observations made by Falnes et al. (2011), who similarly noted the prominent role of mothers-in-law in making health-related decisions within the family unit.

The study revealed that cultural beliefs significantly influence exclusive breastfeeding practices among HIV-positive mothers. These beliefs include concerns that breastmilk naturally makes infants thirsty, restrictions on maternal activities such as sex during breastfeeding or breastfeeding while pregnant, fears that breastmilk spoils if the mother spends time away from the infant, and concerns that infants will reject complementary foods if introduced after six months. Consequently, mothers-in-law often advocate for introducing supplementary foods early to prevent infants from refusing food later on.

Moreover, the study found that HIV-positive postpartum women tend not to practice exclusive breastfeeding due to beliefs that breastmilk induces thirst in infants. Mothers-in-law play a role in encouraging these practices, advising their daughters-in-law to give water to the child. Additionally, regional factors such as sunny weather conditions also contribute to the encouragement of giving water to infants in the study area. These findings are consistent with research indicating that the Upper East Region, where the study was conducted, has a low rate of exclusive breastfeeding (Kubuga & Tindana, 2023). Despite WHO guidelines recommending exclusive breastfeeding for infants exposed to HIV (WHO, 2016), the study highlights that this practice is not widely adopted among HIV-positive pregnant and postpartum women in the region**.**

The findings of the study align with recent reports by Kyei-Author (2021) and Kubuga and Tindana (2023), indicating a decline in exclusive breastfeeding practices. In response, the Ghana Health Service and Ministry of Health are collaborating with WHO and UNICEF to promote awareness about the importance of exclusive breastfeeding. Additionally, the study supports WHO's stance

on breastmilk as the optimal food for infants due to its safety, hygiene, antibodies that protect against childhood illnesses, and nutritional completeness in the first two years of life. However, despite these benefits, a significant proportion of infants do not receive exclusive breastfeeding for the recommended six months.

Furthermore, the findings reaffirm that traditional expectations, particularly those of mothers-in-law, often conflict with recommended infant feeding practices for HIV-positive mothers, such as exclusive replacement feeding or exclusive breastfeeding (Falnes et al., 2011). This contrasts with the UNAIDS (2019) report, which suggests that adherence to infant feeding guidelines may be influenced by male family members, highlighting varied influences on maternal decisions regarding infant feeding practices.

The study findings highlight several factors influencing exclusive breastfeeding practices among HIV-positive mothers, including cultural attitudes and social norms. These norms, such as the use of herbs and liquid foods for infants, often discourage exclusive breastfeeding. Additionally, the educational level of HIV-positive mothers plays a significant role, with those having higher education levels more likely to practice exclusive breastfeeding compared to those with lower education levels, who often receive advice against it from family members. This finding corroborates existing literature indicating that mothers with elementary education or higher are more likely to engage in exclusive breastfeeding (Laksono et al., 2021; Gabriel et al., 2023).

The study underscores the importance of exclusive breastfeeding in low-income communities for HIV-positive mothers, as it reduces the risk of mother-to-child transmission (MTCT) of HIV via breast milk. Early weaning at six months is recommended as a strategy to mitigate this risk; however, cultural norms favouring extended breastfeeding may contradict this practice and

increase the risk of MTCT. The WHO (2017) similarly emphasizes that cultural norms significantly influence exclusive breastfeeding practices, potentially exposing children born to HIV-positive mothers to HIV infection despite being born HIV-negative. This suggests that effective PMTCT intervention programmes should be culturally sensitive and aligned with local practices to promote exclusive breastfeeding and reduce the risk of HIV transmission through breastfeeding.

Food security

The study highlighted food security as a critical factor influencing exclusive breastfeeding practices among participants. Participants consistently emphasized the importance of adequate food intake for successful exclusive breastfeeding. Many reported experiencing food insecurities, which directly impacted their ability to produce sufficient breast milk for their infants. They expressed a belief that inadequate food intake led to insufficient breast milk production, making exclusive breastfeeding challenging. Participants conveyed that if they had access to enough food, they believed they could produce adequate breast milk to exclusively breastfeed their babies. However, due to perceived or actual food shortages, they often found themselves unable to rely solely on breast milk and resorted to supplementing their infants' diets with other foods and water. This finding underscores the need for comprehensive support systems that address food security issues alongside breastfeeding promotion initiatives to improve maternal and child health outcomes.

The study confirmed that HIV-positive mothers often reported insufficient breastmilk production, attributed in this current study to inadequate nutrition as a barrier to optimal breastfeeding (Marinda et al., 2017). However, it contradicted previous literature suggesting that challenges in

breastfeeding compliance were also due to factors like lack of community support and breast infections from poor breastfeeding practices linked to infants' oral health issues (Marinda et al., 2017; Koffi et al., 2023).

Participants in the study described using *zoomkoom*, a local drink made from millet rich in vitamin C, as a substitute for breastmilk when they lacked food. They reported feeding *zoomkoom* to their crying babies, noting that it stimulated breastmilk production. These findings parallel previous research indicating that women give birth at home due to access to culturally accepted foods like *zoomkoom* (Adatara et al., 2019). The participants emphasized that food availability significantly influences their ability to practice exclusive breastfeeding and adhere to medications. This similarity suggests continuity in findings within the same study settings.

The study highlighted that a significant barrier to exclusive breastfeeding in the community was food insecurity, which healthcare providers and lactating mothers both identified as a critical issue. Mothers reported starting exclusive breastfeeding but struggled to continue due to insufficient food to produce adequate breastmilk, leading some to switch to mixed feeding. These findings align with previous research linking food security to exclusive breastfeeding practices among HIV-positive mothers (Samburu et al., 2021; Agyekum et al., 2022). However, contrary to some literature, the study did not find support for factors such as poor policy dissemination, gaps in knowledge, misunderstandings about exclusive breastfeeding, lack of counselling, or stigma related to HIV transmission fears influencing non-compliance with exclusive breastfeeding (Ahmad et al., 2012; Samburu et al., 2021; AlQurashi et al., 2023; Meyer et al., 2024; Mwakawanga et al., 2024).

Conclusions

The study found that exclusive breastfeeding practices among HIV-positive pregnant and postpartum women in the Talensi and Bongo District Hospitals were significantly influenced by cultural practices, traditional infant feeding methods, the customary role of mothers-in-law, and food security. These diverse obstacles require a comprehensive approach to social interventions to achieve zero HIV mother-to-child transmission (MTCT). The findings suggest incorporating self-efficacy theories into individualized interventions to improve mothers' breastfeeding skills and confidence, considering social and environmental factors. Additionally, policies should promote culturally adaptable, community-based breastfeeding programmes in rural areas. Community education and durbars on PMTCT are also needed to raise awareness and acceptance of exclusive breastfeeding among family members in these areas. The PMTCT programme should collaborate with community stakeholders to promote exclusive breastfeeding among postpartum women. It is recommended that the PMTCT programme include a food package for low-income women. Social interventions aimed at eliminating MTCT should be tailored to incorporate the cultural and traditional role of mothers-in-law.

The study provides a comprehensive understanding of contextual barriers, offering a detailed understanding of the multifaceted challenges mothers face in adhering to recommended breastfeeding practices. By highlighting cultural practices like traditional infant feeding methods and the influential role of mothers-in-law, the study provides valuable insights into deeply rooted beliefs and behaviours shaping breastfeeding decisions within these communities. This cultural context enriches the study by revealing factors that may not be evident in other settings.

Moreover, the study's findings are highly relevant to global health goals, particularly those focused on eliminating mother-to-child HIV transmission through breastfeeding interventions. By aligning with these objectives, the study contributes to broader efforts in global health strategies,

underscoring the importance of culturally sensitive approaches for achieving positive maternal and child health outcomes. The application of the theory of planned behaviour enhances the study's theoretical framework, offering a structured analysis of health beliefs and behaviours related to breastfeeding. This theoretical approach not only deepens the understanding of factors influencing breastfeeding practices but also enhances the study's utility in informing public health interventions and policies.

Additionally, the study proposes practical policy recommendations for inclusive Prevention of Mother-to-Child Transmission (PMTCT) programmes and community-based interventions. These recommendations, rooted in the study's findings, aim to effectively address local contexts and barriers. This approach holds promise for potentially improving breastfeeding practices among HIV-positive mothers in similar rural settings.

Limitations of the Study

The study offers valuable insights but also presents several limitations. Firstly, its findings may lack generalizability due to the specific cultural and social contexts of the rural study area, limiting their applicability to different regions or populations with distinct sociocultural dynamics. Moreover, the study's focus on rural communities and specific healthcare facilities introduces potential sampling bias, potentially neglecting diverse perspectives beyond these settings.

Additionally, reliance on self-reported data from participants may introduce biases like social desirability or recall bias, impacting the accuracy of reported breastfeeding practices and beliefs. Furthermore, the dynamic nature of health policies and cultural practices means the study's findings may have limited temporal relevance over time as policies and societal norms evolve. Lastly, while the qualitative insights are valuable, the absence of quantitative data on the

prevalence and impact of cultural beliefs and practices related to breastfeeding limits the study's depth in assessing broader population trends and quantitative outcomes.

Availability of data

The study's data cannot be publicly shared due to containing sensitive, identifiable patient information. Access to the dataset is available to qualified parties meeting criteria for confidential data access through the corresponding author.

Disclosure of conflict of interest

The authors declare no conflict of interest

Authors contributions

The corresponding author conceptualises, collects, analyses, and interprets data. The work was overseen by three supervisors, and the manuscript was approved for submission and publication.

There is no funding regarding this manuscript

References

Abdul, A., Smith, B., & Jones, C. (2021**).** *Effects of Maternal Nutrition on Pregnancy and Breastfeeding Outcomes*. *Journal of Maternal Health,* 15(4), 234-250

Abebe, H., Beyene, G. A., & Mulat, B. S. (2021). Harmful cultural practices during perinatal period and associated factors among women of childbearing age in Southern Ethiopia: Community based cross-sectional study. *PloS one, 16(7), pp 30-45.*

Adatara, P., Strumpher, J., Ricks, E., & Mwini-Nyaledzigbor, P. P. (2019). Cultural beliefs and practices of women influencing home births in rural Northern Ghana. *International journal of women's health, 11, 353.*

Agyekum, M. W., Codjoe, S. N., Dake, F. A., & Abu, M. (2022). Enablers and inhibitors of exclusive breastfeeding: perspectives from mothers and health workers in Accra, Ghana. *International Breastfeeding Journal, 17(1), 1-15.*

Ahmad, K. Y., Page, A., Arora, A., & Ogbo, F. A. (2019). Trends and determinants of early initiation of breastfeeding and exclusive breastfeeding in Ethiopia from 2000 to 2016. *International breastfeeding journal*, *14*, 1-14.

Alhazmi, A. A., & Kaufmann, A. (2022). Phenomenological qualitative methods applied to the analysis of cross-cultural experience in novel educational social contexts. *Frontiers in Psychology, 13, 1495*

AlQurashi, A., Wani, T., Alateeq, N., & Heena, H. (2023, March). Effect of Counselling Service on Breastfeeding Practice among Saudi Mothers. In Healthcare (Vol. 11, No. 6, p. 878). MDPI.

Amedeo Giorgi (2009): Developed the "descriptive phenomenological method" in psychology

Andina Díaz, E., & Siles González, J. (2020). Cultural Care of Pregnancy and Home Birth: *An Application of the Sunrise Model.*

Choi, Y., & Suh, K. H. (2022). Verifying the usefulness of the theory of planned behaviour model for predicting illegal use of online content: the role of outcome expectancies and social loafing. *BMC psychology, 10(1), 263*.

Colaizzi, P. F. (1978). *Psychological Research as the Phenomenologist Views It*. In R. S. Valle & M. King (Eds.), Existential-Phenomenological Alternatives for Psychology (pp. 48-71). Oxford University Press, New York.

Creswell, J. W. (2013). *Qualitative inquiry and research design: Choosing among five approaches*. Sage.

Creswell, John W. (1998). Qualitative inquiry and research design: Choosing among five traditions. Thousand Oaks, CA: Sage.

Falnes, E. F., Moland, K. M., Tylleskär, T., de Paoli, M. M., Msuya, S. E., & Engebretsen, I. M. (2011). “It is her responsibility”: partner involvement in prevention of mother to child transmission of HIV programmes, northern Tanzania. *Journal of the International AIDS Society, 14(1), 21-21*.

Fassinou, L. C., Ouoba, J., Ngwasiri, C., Romba, I., Zoungrana-Yameogo, W. N., Bakiono, F., & Kirakoya-Samadoulougou, F. (2023). Uptake of prevention of mother-to-child transmission cascade services in Burkina Faso between 2013 and 2020: are we on the right track? *BMC Women's Health, 23(1), 126*

Fauk, N. K., Ward, P. R., Hawke, K., & Mwanri, L. (2021). HIV stigma and discrimination: perspectives and personal experiences of healthcare providers in Yogyakarta and Belu, *Indonesia. Frontiers in medicine, 8, 625787.*

Gabriel, M. Á. M., Lozoya, S. M., Ibarra, S. D. L. H., Comeche, L. D., Carrasco, E. G., Mallada, P. L.& Vicente, A. R. (2023). Association Of the Presence of a Covid-19 Infection at The Time of Birth and The Rates of Exclusive Breastfeeding Upon Discharge in Bfhi Hospitals: *A Multicentre, Prospective Cohort Study.*

Giorgi, A. (2009). The descriptive phenomenological method in psychology: A modified Husserlian approach. Duquesne University Press.

Godin, G., & Kok, G. (1996). The theory of planned behaviour: a review of its applications to health-related behaviours. American journal of health promotion, 11(2), 87-98.

Heuckmann, B., Hammann, M., & Asshoff, R. (2019). Advantages and disadvantages of modelling beliefs by single item and scale models in the context of the theory of planned behaviour. *Education Sciences, 9(4), 268.*

Kameni, B. S., Nansseu, J. R., Bigna, J. J., Tatah, S. A., & Seyler, C. (2022). Evaluation of strategies for the prevention of mother-to-child transmission of HIV in Cameroon: a mixed qualitative and quantitative cross-sectional analysis from the Adamawa region of Cameroon. *BMC Public Health, 22(1), 2459.*

Kassie, S. Y., Chereka, A. A., & Damtie, Y. (2023). Systematic review and meta-analysis of knowledge on PMTCT of HIV/AIDS and Association factors among reproductive age women in Ethiopia, 2022. *BMC Infectious Diseases, 23(1), 1-9.*

Khani Jeihooni, A., Jormand, H., Saadat, N., Hatami, M., Abdul Manaf, R., & Afzali Harsini, P. (2021). The application of the theory of planned behaviour to nutritional behaviours related to cardiovascular disease among the women. *BMC Cardiovascular Disorders, 21(1), 1-11.*

Koffi, I., Essis, E. M. L., Bamba, I., Assi, K. R., Konan, L. L., & Aka, J. (2023). Factors associated with exclusive breastfeeding of children under six months of age in Cote d'Ivoire. *International Breastfeeding Journal, 18(1), 1-14*

Kubuga, C. K., & Tindana, J. (2023). Breastfeeding environment and experiences at the workplace among health workers in the Upper East Region of Ghana. *International Breastfeeding Journal, 18(1), 1-13.*

Kyei-Arthur, F., Agyekum, M.W. and Afrifa-Anane, G.F., 2021. The association between paternal characteristics and exclusive breastfeeding in Ghana. *PloS one, 16(6), p.e0252517*
Laksono, A. D., Wulandari, R. D., Ibad, M., & Kusrini, I. (2021). The effects of mother's education on achieving exclusive breastfeeding in Indonesia. *BMC Public Health, 21(1), 1-6.*

Lincoln, Y. S., & Guba, E. G. (1985). *Naturalistic Inquiry*. Sage Publications, Newbury Park, CA.

Malindi, F. C., Maputle, M. S., & Nemathaga, L. H. (2022). Grandmothers' influence on the implementation of PMTCT interventions within 6 weeks at rural areas in Limpopo province: A qualitative study. *Journal of HIV/AIDS & Social Services, 21(1), 1-16.*

Marinda, P., Chibwe, N., Tambo, E., Lulanga, S., & Khayeka Wandabwa, C. (2017). Challenges and opportunities of optimal breastfeeding in the context of HIV option B+ guidelines. *BMC Public Health, 17(1), 1-13.*

Max Van Manen (1996). Researching Lived Experience: Human Science for an Action Sensitive Pedagogy. Ontario, Canada: The Althouse Press.

Meyer, H., Lee, N., George, K., & Kearney, L. (2024*).* Factors influencing midwives' intentions to facilitate normal physiological birth: A qualitative study. *Women and Birth, 37(4), 101617.*

Mohammed, S., Yakubu, I., Fuseini, A. G., Abdulai, A. M., & Yakubu, Y. H. (2023). Systematic review and meta-analysis of the prevalence and determinants of exclusive breastfeeding in the first six months of life in Ghana. *BMC Public Health, 23(1), 920.*

Moustakas, C. (1994). *Phenomenological research methods*. Sage.

Mwakawanga, D. L., Chen, S., Mwilike, B., Lyimo, A. A., Hirose, N., & Shimpuku, Y. (2024). Association between decision-making during pregnancy and woman-centred care among Tanzanian pregnant women: A cross-sectional survey. *Women and Birth*, *37*(4), 101615.

Naidoo, K., Hoque, M., Buckus, S., Hoque, M., & Jagernath, K. (2023). Prevention-of-mother-to-child-transmission (PMTCT) program outcomes in South Africa in the pre-COVID and COVID eras. *BMC Public Health, 23(1), 1-9.*

Rodríguez, G., Pérez, J., Cueva, S., & Torres, R. (2017). A framework for improving web accessibility and usability of Open Course Ware sites. *Computers & education, 109, 197-215.*

Samburu, B. M., Kimiywe, J., Young, S. L., Wekesah, F. M., Wanjohi, M. N., Muriuki, P., & Kimani-Murage, E. W. (2021). Realities and challenges of breastfeeding policy in the context of HIV: a qualitative study on community perspectives on facilitators and barriers related to breastfeeding among HIV positive mothers in Baringo County, Kenya*. International breastfeeding journal, 16(1), 1-13.*

Shetty, K. A., (2013). Epidemiology of HIV infection in women and children: a global perspective. *Current HIV research, 11(2), pp.81-92.*

Shi, H., Wang, J., Huang, R., Zhao, J., Zhang, Y., Jiang, N.& Xu, X. (2021). Application of the extended theory of planned behaviour to understand Chinese students' intention to improve their oral health behaviours: a cross-sectional study. *BMC Public Health, 21, 1-9.*

Tesfaye, M., Solomon, N., Getachew, D., & Biru, Y. B. (2022). Prevalence of harmful traditional practices during pregnancy and associated factors in Southwest Ethiopia: a community-based cross-sectional study. *BMJ open, 12(11), 15-20*

UNAIDS. (2019). *Global AIDS Update 2019: Communities at the Centre*. Joint United Nations Programme on HIV/AIDS (UNAIDS), Geneva, Switzerland.

UNICEF. (2020). *State of the World's Children 2019: Children, Food and Nutrition – Growing Well in a Changing World*. New York: UNICEF.

World Health Organization**.** (2016). *World Health Statistics 2016: Monitoring Health for the SDGs, Sustainable Development Goals*. Geneva: World Health Organization.

World Health Organization. (2017). *WHO report on the global tobacco epidemic, 2017:* monitoring tobacco use and prevention policies. World Health Organization

World Health Organization. (2020*). Mental health and psychosocial considerations during the COVID-19 outbreak*, 18 March 2020 (No. WHO/2019-nCoV/Mental Health/2020.1). World Health Organization

World Health Organization. (2020*). Mental health and psychosocial considerations during the COVID-19 outbreak*, 18 March 2020 (No. WHO/2019-nCoV/Mental Health/2020.1). World Health Organization.

World Health Organization. (2022). *World Health Statistics 2022: Monitoring Health for the SDGs, Sustainable Development Goals*. Geneva: World Health Organization.
