# Supplementary material for: Breastfeeding complexities and sociocultural barriers in the context of preventing perinatal transmission of HIV: A descriptive phenomenology in Northern Ghana
Source: PLoS One. 2025 Jul 1;20(7):e0327353. doi: 10.1371/journal.pone.0327353 (PMC12212534; doi:10.1371/journal.pone.0327353)
Supplement: Attachment — Submitted filename: Comments_Indigenous breastfeeding knowledge.docx [file pone.0327353.s002.docx]

Comments

This study is about barriers to exclusive breastfeeding and replacement feeding and provides interesting and valuable insights into the situation as it exists in Ghana. The authors are commended for conducting this important study.

Below please receive my minor inputs

1. There are two titles which present different variables, though related. However, this is confusing in light of the contents of the article and the conclusions drawn. My input was based on the title: ‘Breastfeeding complexities and sociocultural barriers in the context of preventing perinatal transmission of HIV’. This is theme that seemed to run through the article. This is however in conflict with the title of the manuscript shared by the journal: Indigenous Breastfeeding Beliefs and Practices in Rural Northern Ghana: A Study Among HIV-positive Mothers

The authors are advised to explicitly state the title of this manuscript.

**Abstract:**

***Purpose:***

2. Replace replacement breastfeeding with replacement *feeding*. Please effect this change throughout the manuscript where replacement feeding is referred to as replacement breastfeeding.

3. The authors are advised to include a phrase ‘as an alternative where feasible’ between ‘excusive breastfeeding and replacement feeding’ in the sentence to make it clear that replacement feeding is not equated to exclusive breastfeeding. Or any phrase of the authors choice

***Methods***

Describe the population and sampling method

***Results:***

4. Remove the discussion about foremilk. Include only the themes and (subthemes, if the word count allows).

5. Sentence starting with ‘factors influencing exclusive breastfeeding… ‘ . Mention those factors and remove all points that relate to the discussion of findings.

***Keywords:***

6. Suggestion to include barriers

**Introduction**

7. After the 1^st^ paragraph, please describe the PMTCT programme. As well take the reader through the situation before PMTCT and its success and perhaps gaps as an introduction to the study. The authors are also advised to give some background related to the benefits of exclusive breastfeeding within the HIV context and replacement feeding as an alternative. Inclusion of some actual studies would add some depth to the introduction.

The use of the term ‘further’ in the sentence starting with: ‘cultural perceptions of breastfeeding among pregnant and postpartum women’ gives the impression that other factors have been discussed already. Please revise the use of the term ‘further’.

The authors are advised to make reference to existing studies related to breastfeeding practices among HIV positive women elsewhere and briefly outline some of those practices. The last paragraph before the theoretical framework: please include the perinatal period as well, as implicated within the manuscript’s contents.

The use of the term adherence in the last paragraph of the introduction may need to be revised or be extended by including barriers as well

**8. Theoretical framework**

The discussion on the theoretical framework can be removed. The framework details the phenomenological framework, which is not the main focal point of this study. More attention could rather be paid to the introduction and background to this study.

**Research methods**

***Study setting and design***

9. Explain what ‘traditional marketing’ refers to.

10. It is not clear how the teenage pregnancy rates relate to this manuscript. Consider revising its inclusion or alternatively, expand on its significance. Were the clinics only servicing HIV positive patients?

11. Again there is extensive description of the descriptive phenomenology’ . The authors are advised to summarise the concept within the design section and move aspects related to trustworthiness and analysis to the relevant sections of the manuscript.

12. Revise the data collection period included in the study. The period is mentioned as April – Dec, whereas it is mentioned as March – Sept in the data collection section.

***Participants and recruitment criteria***

13. In exploring the registers, explain how far long back the authors went in identifying the respondents for inclusion into the study (number of years/months).

14. Please explain how the participants were accessed. Were they called on their phones? Visited in their homes? Recruited on the day of consultation?

15. Explain how the sample size of 32 was decided upon.

***Data collection tool and procedure***

16. Explain what is meant by ‘data collection was carried out systematically……

17. It is not clear who was subjected to informal interviews and who took part in in-depth interviews, whether the same tool was used or not or the reasons for using different approaches to data collection.

18. There are a number of statements that need to be referenced as they represent other author’s ideas.

19. Did both the informal and in-depth interviews last for 45 -60 min?

20. Expand on the tool a bit- how it was designed and the actual questions included in the interview guide or themes

21. Explain whether a co-coder was involved or not

22. Explain how data was analysed: whether inductive, deductive or abductive?

***Ethical statement***

23. Remove ‘this PhD thesis…

**Results**

24. Suggestion to present the sociodemographic data in a Table format

25. It is not clear how the authors determined the factors contributing to MTCT mentioned under the sociodemographic data (refusal to attend antenatal care, etc.) since no statistical tests were run, unless mothers explicitly said so. Please revise.

***Presentation of results:***

26.Within the first sentence in this section, the authors refer to results related to breastfeeding practices and nothing is mentioned about the barriers as per the title (this raises confusion as this very statement relates to one of the title and not the title that seems to be aligned with the current manuscript). The authors are advised to relook the terminologies used, or otherwise explain links between practice and attitudes (how one was derived from the others, if this was the case).

The authors are advised to compare the themes outlined here as opposed to the ones mentioned in the introduction. Please align the two sections. E.g foremilk being perceived as toxic, being mentioned in the abstract and not the results section, etc. (It would be useful to include the questionnaire as an appendix. This would assist in reviewing the themes presented).

27. *Breastfeeding as a long-standing practice.* The use of the term mothers is suggested, instead of HIV positive mothers.

28. *Traditional infants water feeding as a cultural practice.* Use the term forcefully instead of forcibly. Also use the correct noun instead of **‘it’** in the phrase: ‘Beliefs **it** improves infants health’.

29*. Influence of older women in the family.* Do the mothers-in-law imply that exclusive breastfeeding denies the child to get employment later in life? Please explain.

30. *Challenges in food availability:* I am checking if zoomkoom is made from water or fruit juice, in light of the fact that it is high in vitamin C? please verify or ignore if correct. The authors are advised to relook into the statement that zoomkoos serves as breastmilk, rather use the term ‘lactogogue’ when referring to mothers, maybe?

**Discussion**

31. The discussion and the conclusion sections are too long. The authors are advised to be succinct and to the point.

32. New results are presented in this section, there is a bit of repetition and the authors are advised to rework the sections
