# Supplementary material for: Breastfeeding complexities and sociocultural barriers in the context of preventing perinatal transmission of HIV: A descriptive phenomenology in Northern Ghana
Source: PLoS One. 2025 Jul 1;20(7):e0327353. doi: 10.1371/journal.pone.0327353 (PMC12212534; doi:10.1371/journal.pone.0327353)
Supplement: Attachment — Submitted filename: Responses to Reviewers-Table.docx [file pone.0327353.s004.docx]

**Repones to Reviewers’ Comments**

| **Reviewer # 1 & 2 Comments** | **Response** |
| --- | --- |
| **Title**  1. There are two titles which present different variables, though related. However, this is confusing in light of the contents of the article and the conclusions drawn. My input was based on the title: ‘Breastfeeding complexities and sociocultural barriers in the context of preventing perinatal transmission of HIV’. This is theme that seemed to run through the article. This is however in conflict with the title of the manuscript shared by the journal: Indigenous Breastfeeding Beliefs and Practices in Rural Northern Ghana: A Study Among HIV-positive Mothers The authors are advised to explicitly state the title of this manuscript. | The title has been standardized to ensure clarity and consistency. The chosen title is: “*Breastfeeding complexities and sociocultural barriers in the context of preventing perinatal transmission of HIV: A descriptive phenomenology in Northern Ghana*." **See Title page 1** |
| **Abstract**  **Purpose**  **2**. Replace replacement breastfeeding with replacement feeding. Please effect this change throughout the manuscript where replacement feeding is referred to as replacement breastfeeding. | The term *"replacement breastfeeding"* has been replaced with *"replacement feeding"* throughout the manuscript as suggested. **See abstract page 1** |
| **3**. The authors are advised to include a phrase ‘as an alternative where feasible’ between ‘exclusive breastfeeding and replacement feeding’ in the sentence to make it clear that replacement feeding is not equated to exclusive breastfeeding. Or any phrase of the authors choice. | The phrase *"as an alternative where feasible"* has been included between *"exclusive breastfeeding and replacement feeding"* for clarity. **See abstract page 1** |
| **Methods**  Describe the population and sampling method  **4**. Remove the discussion about foremilk. Include only the themes and (subthemes, if the word count allows). | Population and sampling method have been described as suggested.  The discussion about foremilk has been removed, and only the themes and subthemes are presented. **See abstract page 1** |
| **5**. Sentence starting with ‘factors influencing exclusive breastfeeding… ‘. Mention those factors and remove all points that relate to the discussion of findings. | The sentence has been revised to explicitly mention the factors influencing exclusive breastfeeding, and points unrelated to findings have been removed. **Page 1** |
| **Keywords**  **6.** Suggestion to include barriers in the keywords. | The keyword *"barriers"* has been added to the list of keywords**. Page 1** |
| **Introduction**  **7.** After the 1st paragraph, please describe the PMTCT programme. As well take the reader through the situation before PMTCT and its success and perhaps gaps as an introduction to the study. The authors are also advised to give some background related to the benefits of exclusive breastfeeding within the HIV context and replacement feeding as an alternative. Inclusion of some actual studies would add some depth to the introduction. The use of the term ‘further’ in the sentence starting with: ‘cultural perceptions of breastfeeding among pregnant and postpartum women’ gives the impression that other factors have been discussed already. Please revise the use of the term ‘further’. The authors are advised to make reference to existing studies related to breastfeeding practices among HIV positive women elsewhere and briefly outline some of those practices. The last paragraph before the theoretical framework: please include the perinatal period as well, as implicated within the manuscript’s contents. The use of the term adherence in the last paragraph of the introduction may need to be revised or be extended by including barriers as well. | The PMTCT programme has been described in detail, including the pre- and post-PMTCT phases. The use of *"further"* has been revised, and references to existing studies on breastfeeding practices among HIV-positive women have been added. The perinatal period and barriers have also been included as suggested. **See page 2** |
| **Theoretical framework**  **8**. The discussion on the theoretical framework can be removed. The framework details the phenomenological framework, which is not the main focal point of this study. More attention could rather be paid to the introduction and background to this study. | The theoretical framework has been removed, and the introduction and background have been expanded for greater depth and relevance to the study. **See page 5** |
| **Research methods**  **Study setting and design**  **9**. Explain what ‘traditional marketing’ refers to. | The term *"traditional marketing"* has been reframed and clarified. **See page 7-8** |
| **10**. It is not clear how the teenage pregnancy rates relate to this manuscript. Consider revising its inclusion or alternatively, expand on its significance. Were the clinics only servicing HIV positive patients? | The reference to teenage pregnancy rates has been revised for relevance. **See page 6-7** |
| **11**. Again, there is extensive description of the descriptive phenomenology’. The authors are advised to summarise the concept within the design section and move aspects related to trustworthiness and analysis to the relevant sections of the manuscript. | The description of descriptive phenomenology has been summarized within the design section, with aspects of trustworthiness and analysis moved to their appropriate sections. **See page 7 and 14 respectively** |
| **12**. Revise the data collection period included in the study. The period is mentioned as April – Dec, whereas it is mentioned as March – Sept in the data collection section. | The data collection period has been corrected and standardized throughout the manuscript.  **See page 8** |
| **Participants and recruitment criteria**  **13**. In exploring the registers, explain how far long back the authors went in identifying the respondents for inclusion into the study (number of years/months). | The timeline for reviewing the registers has been clarified, with number of months. **See page 8-9** |
| **14.** Please explain how the participants were accessed. Were they called on their phones? Visited in their homes? Recruited on the day of consultation? | Participants were accessed through clinic visits and follow-ups and conducted during home visits. **See page 8** |
| **15**. Explain how the sample size of 32 was decided upon. | The sample size of 32 was determined based on data saturation, where no new themes emerged after 32 interviews. **See page** **8- 9** |
| **Data collection tool and procedure**  **16.** Explain what is meant by ‘data collection was carried out systematically…… | The phrase *"data collection was carried out systematically"* has been reframed. **See page 8** |
| **17**. It is not clear who was subjected to informal interviews and who took part in in-depth interviews, whether the same tool was used or not or the reasons for using different approaches to data collection. | This has been reframed as interviews were conducted with mothers, using interview guide. **See page 9** |
| **18.** There are a number of statements that need to be referenced as they represent other author’s ideas. | Missing references have been added to properly attribute the original authors' ideas. **See page 9-11** |
| **19**. Did both the informal and in-depth interviews last for 45 -60 min? | The interviews lasted between 45-60 minutes, as now clarified. |
| **20**. Expand on the tool a bit- how it was designed and the actual questions included in the interview guide or themes. | The description of the interview tool has been expanded, including key themes and sample questions. **See page 8-9** |
| **21**. Explain whether a co-coder was involved or not. | A co-coder was involved to ensure data reliability, and this has been clarified. **See page 11** |
| **22.** Explain how data was analysed: whether inductive, deductive or abductive? | The analysis approach was an *inductive thematic analysis.* ***See detail in page 11-12*** |
| **Ethical statement**  **23**. Remove ‘this PhD thesis… | The phrase *"this PhD thesis"* has been removed. |
| **Results**  **24**. Suggestion to present the sociodemographic data in a Table format. | Sociodemographic data has been reformatted into a table for clarity. **See page 15** |
| **25.** It is not clear how the authors determined the factors contributing to MTCT mentioned under the sociodemographic data (refusal to attend antenatal care, etc.) since no statistical tests were run, unless mothers explicitly said so. Please revise. | The factors contributing to MTCT were based on participant statements and not statistical tests. **See detail in page 16** |
| **Presentation of results:**  **26**. With the first sentence in this section, the authors refer to results related to breastfeeding practices and nothing is mentioned about the barriers as per the title (this raises confusion as this very statement relates to one of the titles and not the title that seems to be aligned with the current manuscript). The authors are advised to relook the terminologies used, or otherwise explain links between practice and attitudes. | The results section has been revised to clearly reflect both breastfeeding practices and barriers. **See page 16-22** |
| **27**. Breastfeeding as a long-standing practice. The use of the term mothers is suggested, instead of HIV positive mothers. | The term *"mothers"* has been used instead of *"HIV positive mothers"* for generalization as suggested. **See page 17** |
| 28. Traditional infants water feeding as a cultural practice. Use the term forcefully instead of forcibly. Also use the correct noun instead of ‘it’ in the phrase: ‘Beliefs it improves infants health’. | The term *"forcefully"* has replaced *"forcibly"*, and the noun *"water feeding"* has been used instead of *"it.***" See page 18** |
| **29**. Influence of older women in the family. Do the mothers-in-law imply that exclusive breastfeeding denies the child to get employment later in life? Please explain. | Clarification has been added that mothers-in-law the child by denying him water, not employment outcomes. **See page 19** |
| **30**. Challenges in food availability: I am checking if zoomkoom is made from water or fruit juice, in light of the fact that it is high in vitamin C? please verify or ignore if correct. The authors are advised to relook into the statement that zoomkoom serves as breastmilk, rather use the term ‘lactogogue’ when referring to mothers, maybe? | Clarification has been added that *zoomkoom* is made from millet and water, not fruit juice, and the term *"zoomkoom"* has been used appropriately. The correct term is *lactogogue* and not breastmilk. Thank you for the suggestion |
| **Discussion**  **31**. The discussion and the conclusion sections are too long. The authors are advised to be succinct and to the point. | The discussion and conclusion sections have been condensed for clarity and brevity. **See page 22-29** |
| **32**. New results are presented in this section, there is a bit of repetition and the authors are advised to rework the sections. | Repetition has been removed, and new results have been cheeked to align with the results presented. **See page 22-99** |
